# The Role of Peritoneal Alternatively Activated Macrophages in the Process of Peritoneal Fibrosis Related to Peritoneal Dialysis

**DOI:** 10.3390/ijms140510369

**Published:** 2013-05-17

**Authors:** Jie Wang, Zong-Pei Jiang, Ning Su, Jin-Jin Fan, Yi-Ping Ruan, Wen-Xing Peng, Ya-Fang Li, Xue-Qing Yu

**Affiliations:** 1 Department of Nephrology, the First Affiliated Hospital, Sun Yat-sen University, Guangzhou 510080, China; 2 Key Laboratory of Nephrology, Ministry of Health, the First Affiliated Hospital, Sun Yat-sen University, Guangzhou 510080, China; E-Mails: wangjiedemuma@163.com (J.W.); jx.home@medmail.com.cn (Z.-P.J.); gysuning@163.com (N.S.); fanjinj@mail.sysu.edu.cn (J.-J.F.); tyryp@163.com (Y.-P.R.); pwx005@126.com (W.-X.P.); liyafang.259@163.com (Y.-F.L.); 3 Department of Nephrology, the Sixth Affiliated Hospital, Sun Yat-sen University, Guangzhou 510655, China

**Keywords:** peritoneal dialysis, peritoneal fibrosis related to PD, alternatively activated macrophages, liposome-encapsulated clodronate

## Abstract

It has been confirmed that alternatively activated macrophages (M2) participate in tissue remodeling and fibrosis occurrence, but the effect of M2 on peritoneal fibrosis related to peritoneal dialysis (PD) hasn’t been elucidated. This study was therefore conducted to assess the association between M2 and peritoneal fibrosis related to PD. In this study, peritoneal fibrosis was induced by intraperitoneal (i.p.) injection of Lactate-4.25% dialysate (100 mL/kg) to C57BL/6J mice for 28 days, and liposome-encapsulated clodronate (LC, the specific scavenger of macrophages) was used to treat the peritoneal fibrosis mice model by i.p. injection at day 18 and day 21. All animals were sacrificed at day 29. Parietal peritonea were stained with Masson’s trichrome, and the expression of type I collagen (Col-I), fibronectin, mannose receptor (CD206), transforming growth factor beta (TGF-β), chemokine receptor 7 (CCR7), chitinase 3-like 3 (Ym-1) and arginase-1 (Arg-1) was determined by Western blotting, immunofluorescence and quantitative real-time PCR. Our results revealed that peritoneal thickness, Col-I, fibronectin, CD206, TGF-β, Ym-1 and Arg-1 were upregulated in the peritoneal fibrosis mice model, and all of these indexes were downregulated in those treated with LC. Additionally, there was no difference in the level of CCR7 between the model and treatment group. Our study indicated that peritoneal M2 played an important role in the process of peritoneal fibrosis related to PD and might be a potential target for intervention therapy of peritoneal fibrosis.

## 1. Introduction

Since the invention of continuous ambulatory peritoneal dialysis (CAPD), peritoneal dialysis (PD) became universally used in end-stage renal disease (ESRD) patients [1,2].

One of the most important problems is the maintenance of the peritoneal membrane in long-term PD [3]. It is well known that a healthy peritoneum is composed of a single layer of mesothelial cells (MC) resting upon a thin submesothelial compact collagenous zone comprising a few collagen deposition, fibroblasts, macrophages and vessels [2,4,5]. However, long-term PD leads to peritoneal fibrosis characterized by a higher density of blood vessels in the peritoneum, increased thickness of the submesothelial compact zone and vasculopathy, which is related to the loss of peritoneal function and, ultimately, results in treatment failure [6].

Macrophages are the most plastic immune cells distributed widely in organs and tissues, which polarize into distinct populations determined by the microenvironment [7,8]. It is generally accepted that classically activated macrophages (M1) are induced by lipopolysaccharide (LPS) and interferon-γ (IFN-γ), which perform a high expression of inducible nitric oxide synthase (iNOS) and chemokine receptor 7 (CCR7) and secrete lots of pro-inflammatory cytokines involved in tissue damage [7,9–11]. In reverse, exposure to Interleukin-4 (IL-4) and Interleukin-13 (IL-13) stimulates alternative activation of macrophages (M2) characterized by upregulation of the mannose receptor (CD206), transforming the growth factor beta (TGF-β), chitinase 3-like 3 (Ym-1) and activity of arginase-1 (Arg-1), which are considered to participate in tissue repair and fibrosis [8,12,13].

Recently, several studies make a statement that M2 macrophages play an important role in different fibrotic diseases. In addition, the fibrosis can be mitigated along with the depletion or inhibition of M2 [14–19]. Nevertheless, as a major component of the abdominal cavity immune system, the relationship between peritoneal macrophages, especially M2, and peritoneal fibrosis remains uncertain. Therefore, we hypothesize that M2 may play an important role in the process of peritoneal fibrosis related to PD. Thus, the aim of this study is to investigate the accumulation of M2 macrophages in the peritoneal fibrosis mice model and the role of M2 macrophages in the process of peritoneal fibrosis related to PD.

## 2. Results

### 2.1. Alteration of Peritoneum in Peritoneal Fibrosis Mice

**Figure 1:**
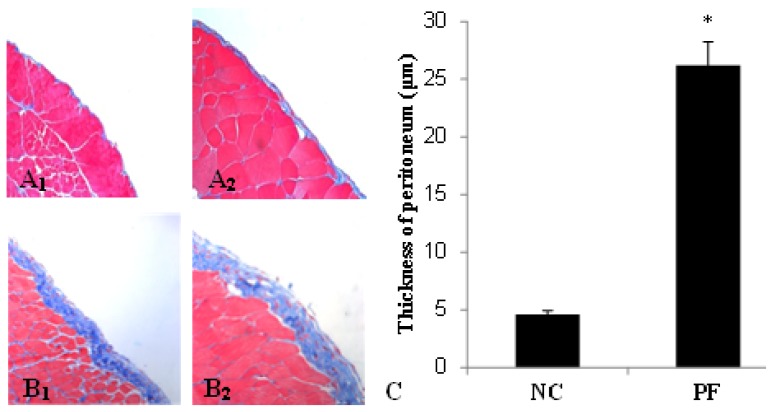
Masson’s trichrome staining of parietal peritoneum slices. (**A_1_**) Normal control (original magnification: ×200); (**A_2_**) Normal control (original magnification: ×400); (**B_1_**) Peritoneal fibrosis (PF) group (original magnification: ×200); (**B_2_**) PF group (original magnification: ×400); (**C**) The thickness of the peritoneum (mean ± SD). The thickness of the peritoneum in the PF group was significantly thicker than that in the NC group. * *p* < 0.05, *vs.* NC group.

Morphologic changes were assessed in tissue sections of the parietal peritoneum with Masson’s trichrome. In peritoneal fibrosis (PF) mice, we observed a loss of mesothelial cells and enlargement of the submesothelial zone with massive cellular infiltration and collagen deposition, while normal control (NC) mice displayed a monolayer of mesothelial cells resting upon a thin submesothelial compact zone (Figure 1A_1_,A_2_,B_1_,B_2_). Accordingly, mice from the PF group showed progressive thickening of peritoneum compared with the NC group (26.2 ± 2.03 *vs.* 4.6 ± 0.34 μm, *p <* 0.05) (Figure 1C).

### 2.2. Macrophage Polarization in PF Mice

**Figure 2:**
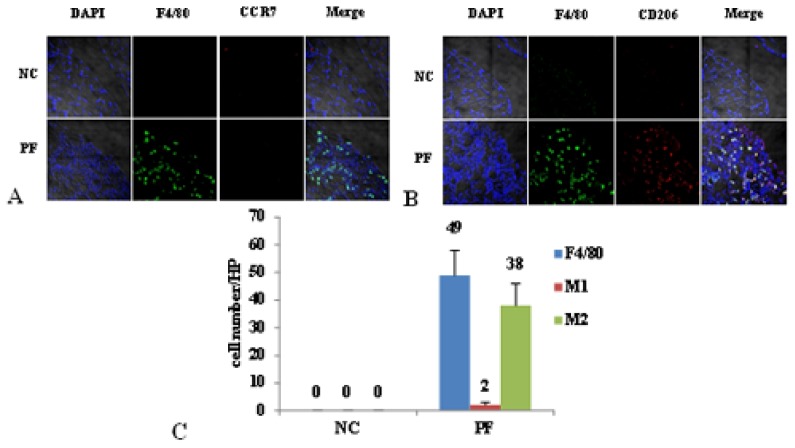
Macrophage polarization in PF mice. (**A**) The number of M1 didn’t change between normal control (NC) and PF mice. Blue corresponds to nuclear staining, green corresponds to F4/80 and red corresponds to CCR7; co-location signals were identified as M1; (**B**) The number of M2 in PF mice markedly increased. Blue corresponds to nuclear staining, green corresponds to F4/80 and red corresponds to CD206; co-location signals were identified as M2; (**C**) The cell count of M1 and M2 in the peritoneum of NC and PF mice.

As shown in Figure 2, F4/80 was used as a maker of macrophages, F4/80 and CD206 double positive cells were defined as M2, while F4/80 and CCR7 double positive cells were considered as M1. There was mainly no macrophage infiltration in the NC group. However, in the PF group, the number of macrophages markedly increased, and most of the macrophages were M2, but few were M1.

### 2.3. Effect of Liposome-Encapsulated Clodronate (LC) on Peritoneal M1 and M2

**Figure 3:**
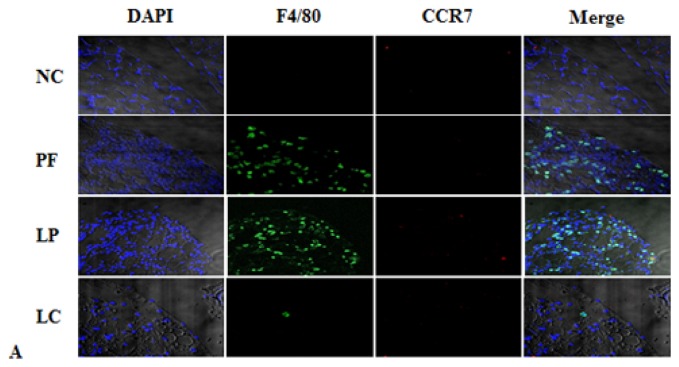

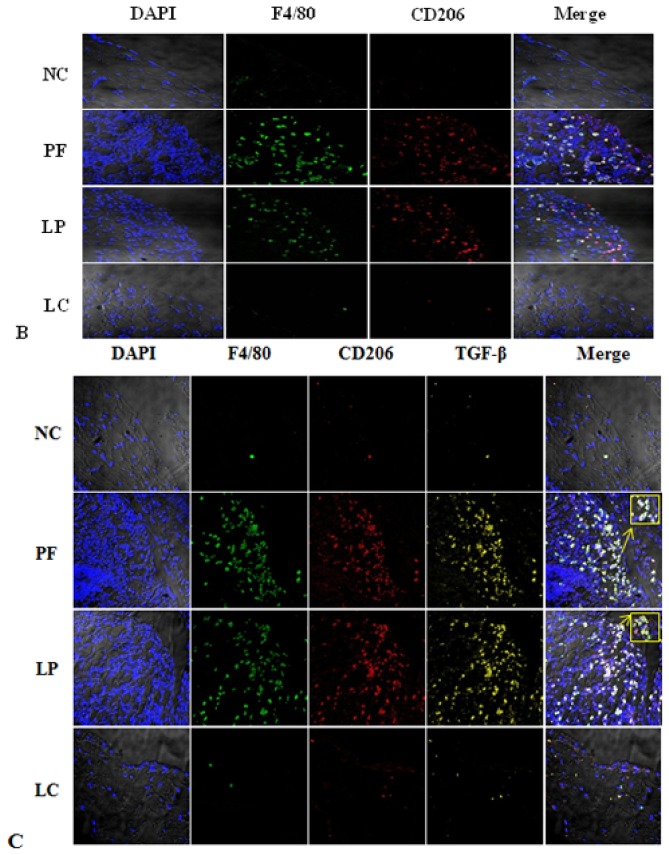
Liposome-encapsulated clodronate (LC) depleted almost all the M2 in the peritoneum without an effect on M1. (**A**) The number of M1 didn’t change among the four groups. Blue corresponds to nuclear staining, green corresponds to F4/80 and red corresponds to CCR7; co-location signals were identified as M1; (**B**) The number of M2 significantly increased after injecting Lactate-4.25% dialysate; liposome-encapsulated PBS (LP) didn’t affect M2 infiltration, but LC depleted peritoneal M2 efficiently. Blue corresponds to nuclear staining, green corresponds to F4/80 and red corresponds to CD206; co-location signals were identified as M2; (**C**) TGF-β was expressed by M2, and LC inhibited the expression of TGF-β by depletion of M2. Blue corresponds to nuclear staining, green corresponds to F4/80, red corresponds to CD206 and yellow corresponds to TGF-β staining.

**Figure 4:**
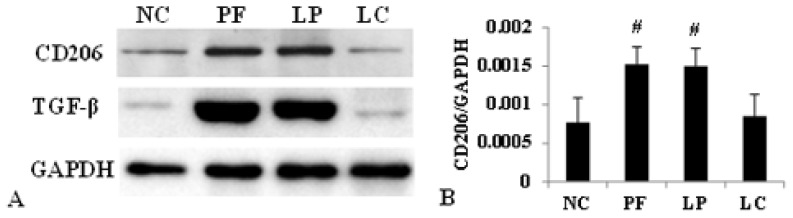

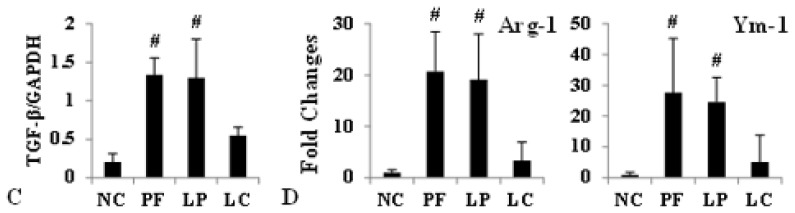
Effect of LC on Lactate-4.25% dialysate-induced overexpression of CD206, TGF-β, Arg-1 and Ym-1. Values were expressed as the mean ± SD. (**A**) The overexpression of CD206 and TGF-β induced by Lactate-4.25% dialysate was evidently downregulated by LC treatment measured by Western blotting; (**B**) The relative protein level of CD206 normalized by GAPDH; (**C**) The relative protein level of TGF-β normalized by GAPDH; (**D**) The mRNA level of Arg-1 and Ym-1 were depressed by LC treatment. ^#^*p* < 0.05, *vs.* NC and LC group.

Immunofluorescence staining revealed that liposome-encapsulated clodronate (LC) injection markedly alleviated M2 infiltration and secretion of TGF-β induced by Lactate-4.25% dialysate (Figure 3B,C), but there were no variations in M1 expression among the four groups (Figure 3A). Furthermore, Western blotting showed that the amounts of CD206 and TGF-β were significantly upregulated in the PF group and the liposome-encapsulated PBS (LP) group, and their expression could also be depressed by LC (Figure 4 A–C). Similarly, LC treatment definitely inhibited the upregulation of Arg-1 and Ym-1 mRNA (Figure 4D).

### 2.4. LC mitigated Peritoneal Fibrosis Related to PD by Depletion of M2 in Peritoneum

**Figure 5:**
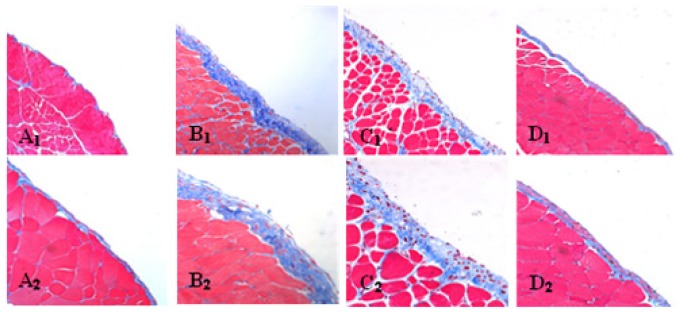
Representative photomicrographs (original magnification: ×200) of Masson’s trichrome staining in the (**A_1_**) NC group, (**B_1_**) PF group, (**C_1_**) LP group and **(D_1_**) LC group. Representative photomicrographs (original magnification: ×400) of Masson’s trichrome staining in the (**A_2_**) NC group, (**B_2_**) PF group, (**C_2_**) LP group and **(D_2_**) LC group.

**Figure 6:**
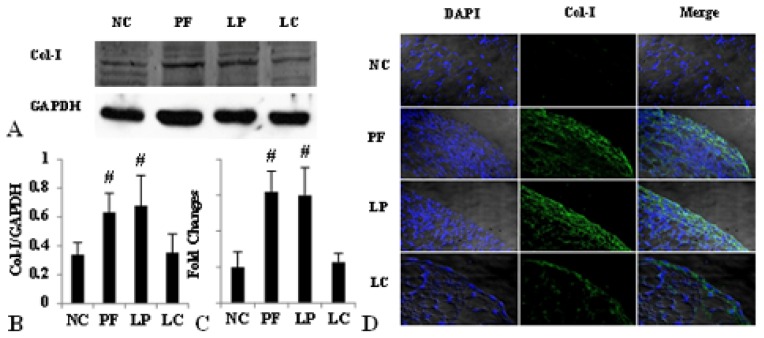
LC depressed Col-I deposition via depletion of peritoneal M2. Values were expressed as the mean ± SD. (**A**) The overexpression of Col-I induced by Lactate-4.25% dialysate was evidently downregulated by LC treatment measured by Western blotting; (**B**) The relative protein level of Col-I normalized by GAPDH; (**C**) The mRNA level of Col-I was depressed by LC treatment; (**D**) Immunofluorescence staining of Col-I in the four groups. Blue corresponds to nuclear staining; green corresponds to Col-I staining. ^#^*p* < 0.05, *vs.* NC and LC group.

**Figure 7:**
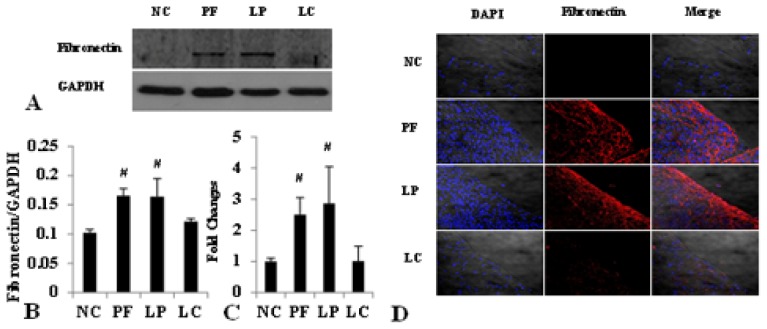
LC depressed both the protein and mRNA level of fibronectin via depletion of peritoneal M2. Values were expressed as the mean ± SD. (**A**) The overexpression of fibronectin induced by Lactate-4.25% dialysate was evidently downregulated by LC treatment measured by Western blotting; (**B**) The relative protein level of fibronectin normalized by GAPDH; (**C**) The mRNA level of fibronectin was depressed by LC treatment; (**D**) Immunofluorescence staining of fibronectin in the four groups. Blue corresponds to nuclear staining, and red corresponds to fibronectin staining. ^#^*p* < 0.05 *vs*. NC and LC group.

Accompanied by the complete depletion of M2 in the peritoneum, the structure of the peritoneum and the extent of fibrosis were improved compared to that in the PF and LP group. As shown in Figure 5, mesothelial cells were arranged compactly and orderly, with fewer deposition of type I collagen (Col-I) after injection of LC, and the thickness of the peritoneum in the LC group was much thinner than that in the PF and LP group (10.0 ± 2.36 *vs.* 26.2 ± 2.03, 10.0 ± 2.36 *vs.* 27.7 ± 4.38, *p <* 0.05) (Table 1). What’s more, both the protein and mRNA level of Col-I and fibronectin were upregulated in PF mice and downregulated, obviously followed by M2 depletion in the LC group examined by Western blotting, immunofluorescence and quantitative real-time PCR (Figures 6 and 7).

## 3. Discussion

In the present study, our data showed that the number of M2 macrophages was significantly increased in the PF group, accompanied with peritoneal fibrosis. And the peritoneal fibrosis was mitigated after depleting M2 by LC. Additionally, there was no difference of the expression of M1 between the PF and LC group.

As the most plastic innate immune cells, the concept of M1 and M2 has obtained considerable ground over the past years, and several studies have demonstrated that M2 macrophages played an important role in fibrotic diseases. In patients with systemic sclerosis, the expression of M2 macrophages was significantly increased and associated with the extent of fibrosis [15]. In patients with idiopathic pulmonary fibrosis (IPF), the human marker of M2, which wasn’t expressed on pulmonary macrophages from healthy volunteers, was shown to be expressed on more than 90% of pulmonary macrophages [19]. In peritoneal dialysis patients, Ahmad *et al.* [20] showed that both the dialysate chemokine (C-C motif) ligand 18(CCL18) and the serum CCL18 were significantly higher in patients who developed encapsulating peritoneal sclerosis (EPS); subsequently, Bellón *et al.* [21] proved that the capacity of M2 macrophages to stimulate fibroblast proliferation was proportional to the mRNA level of CCL18, and they believed that M2 macrophages may participate in human peritoneal fibrosis through the CCL18 production. Especially, it was proven that alternative Kupffer cell activation promoted liver fibrosis without classical activation [22]. In our study, we found that dialysate with a high concentration of glucose stimulated large aggregation of macrophages; most of them displayed an M2 phenotype without classical activation. Besides, as the surface marker and the effector molecule of M2, the upregulation of CD206 and TGF-β was regard as the activation of M2 macrophages.

In order to further investigate the role of M2 in peritoneal fibrosis related to PD, we depleted M2 by LC to observe the improvement of peritoneal fibrosis. LC was developed to deplete macrophages from organs and tissues *in vivo* in the early 1980s [23,24]. In bleomycin-induced lung fibrosis, LC showed an efficient depletion of pulmonary M2 macrophages and mitigated the lung fibrosis, evidenced by the depression of Ym-1 and arginase activity and the related mitigation of lung fibrosis measured by the pulmonary collagen and fibrosis score [19]. Besides, serum amyloid P (SAP) prevented bleomycin-induced lung fibrosis through the inhibition of pro-fibrotic alternative macrophage accumulation, similar to the effect of LC [25]. Although LC targets on all macrophages, Jeike Biewenga *et al.* proved that LC can deplete peritoneal macrophages efficiently for a long time; especially, the effect on M2 was more pronounced and long-lasting, and it might because of the more mature phagocytosis potentiality, which was the most critical factor for LC depletion [26]. What’s more, our previous results showed that i.p. injection of Lactate-4.25% dialysate stimulated massive M2 infiltration without activation of M1; considering all of these, we made the depletion of macrophages to be the equal of depletion of M2 in this study only. In our study, LC injection displayed complete depletion of peritoneal M2 without activation of M1; both the expression of CD206 and TGF-β were depressed after LC treatment. Accompanied with the depletion of peritoneal M2, there was a notable improvement of peritoneal fibrosis. The thickness of the peritoneum was much thinner, the layer of mesothelial cells was protected and the expression of Col-I and fibronectin was reduced. All of these data were in accordance with the studies mentioned previously and indicated that M2 regulated the process of peritoneal fibrosis.

The most intriguing aspect of our study was the data suggesting that during the process of peritoneal fibrosis, peritoneal M2 macrophages depletion suppressed the fibrosis. It highlighted the potential importance of peritoneal M2 macrophages in the process of peritoneal fibrosis related to PD. Besides, unlike other peritoneal fibrosis models induced by chlorhexidine-gluconate (CG), which was neither in accordance with characteristics, such as acidic pH, a high concentration of glucose and glucose degradation products nor used clinically in PD [27,28], we established an animal model by daily i.p. injection of Lactate-4.25% dialysate in order to better simulate the natural history in long-term PD. Furthermore, our data showed a successful PF model characterized by the loss of mesothelial cells in the peritoneum and the significant increase of peritoneal thickness and, also, the overexpression of Col-I and fibronectin.

The mechanism that M2 macrophages promote peritoneal fibrosis hasn’t been discussed in this study. However, it is believed that excessive TGF-β not only increases synthesis of extracellular matrix protein, but also decreases its degradation [29]. Moreover, several studies confirmed that the TGF-β/Smad signaling pathway played an important role in fibrogenesis [30,31]. A previous study in our laboratory showed an inhibition of Smad7 and a markedly upregulated phosphorylation of Smad2/3 in PF mice [32]. Taken together, dialysate containing high glucose induced massive aggregation of peritoneal M2, which, in turn, produced an amount of effector molecules leading to peritoneal fibrosis, which could be suppressed by LC via depletion of M2, and the influence of M2 on peritoneal fibrosis might associate with its effector molecule via TGF-β/Smad signaling pathway. The latter needs to be further confirmed. Since we focused on the relationship between M2 and peritoneal fibrosis, the mechanism by which dialysate with high glucose stimulates infiltration of peritoneal M2 hasn’t been mentioned. Furthermore, the safety and toxic effect of LC haven’t been well studied; thus, there is still a long way to go for clinical use of LC. More studies should be performed in the future.

Our described importance of peritoneal M2 macrophages during peritoneal fibrosis may provide a scientific rationale for peritoneal fibrosis intervention therapy and has wider appeal therapeutically for the treatment of other tissue fibrosis. By enhancing our knowledge of peritoneal fibrosis and peritoneal macrophages, new molecular and pharmaceutical therapeutic approaches may be developed.

## 4. Materials and Methods

### 4.1. Preparation of Liposome-Encapsulated Clodronate and Liposome-Encapsulated Phosphate Buffer

Clodronate was purchased from Shanghai Weijin Biology Company, and LC was prepared according to the standard procedure described previously [26,33]. In general, 86 mg phosphatidylcholine and 8 mg cholesterol were dissolved in a 500 mL round-bottom flask and rotated in low vacuum at room temperature (RT) to form a lacteous film on the inside wall of the flask. For liposome-encapsulated clodronate, 10 mL clodronate solution was added (2.5 g clodronate in 10 mL PBS) into the flask to diffuse the film. After a short sonication for 3 min, the liposome suspension was kept at RT for 1 hour. Then, the LC formed a white band floating on the solution. In order to remove the unencapsulated clodronate, the suspension was centrifuged at 100,000× *g* for 30 min, then washed with sterile phosphate buffer (PBS) three times and finally resuspended with 4 mL sterile PBS. Liposome-encapsulated PBS (LP) preparation was similar to that of LC.

### 4.2. Animal Model and Tissue Preparation

Eight-week-old female C57BL/6J mice were purchased and raised in the Experimental Animal Center of Sun Yat-Sen University. Twenty-four mice were randomly divided into four groups: the PF group (*n* = 6) received daily intraperitoneal (i.p.) injection of Lactate-4.25% dialysate (Baxter, Guangzhou, China) at a dose of 100 mL/kg; the LC group (*n* = 6) was given i.p. injection of Lactate-4.25% dialysate (100 mL/kg) every day and also received i.p. injection of LC (10 mL/kg) at day 18 and day 21; meanwhile, the LP group (*n* = 6) was treated similarly to the LC group, except for the LP injection (10 mL/kg) at day 18 and day 21 instead of LC; and the rest were used as normal control (NC) without any intervention. All animals were sacrificed at day 29. The parietal and visceral layer of peritonea were collected and cut into small pieces, fixed with periodate-lysine-paraform-aldehyde fixative (PLP) at 4 °C overnight and embedded in paraffin; the rest of the visceral peritonea was frozen in liquid nitrogen immediately and stored at −80 °C for future study.

This study has been approved by the ethics committee of the First Affiliated Hospital of Sun Yat-sen University on animal experiments.

### 4.3. Histology

Two micrometers-thick sections of paraffin-embedded parietal peritoneum were cut and stained with Masson’s trichrome. The sections staining with Masson’s trichrome were used to measure the thickness. Firstly, five independent low power (×200) fields in each section were randomly picked under a light microscope (ZEISS Axioplan 2 Imaging, Jena, Germany). Then, five independent sites of each field were randomly picked under a high power (×400) to measure the thickness of the submesothelial compact zone using Axio Vision Rel. 4.5 software (Zeiss, Jena, Germany). Finally, an average thickness of each sample was taken for analysis by SPSS16.0 software (SPSS Inc., Chicago, IL, USA).

### 4.4. Immunofluorescence

Paraffin-embedded visceral peritoneum sections (6 μm) were used to examine expression of TGF-β (CST, Boston, MA, USA) secreted by M2, surface markers of macrophages, such as F4/80 (Abcam, Cambridge, UK), CD206 (Abcam, Cambridge, UK) and CCR7 (Abcam, Cambridge, UK), and the degree of fibrosis with type I collagen (Col-I, Southern Biotech, Birmingham, AL, USA) and fibronectin (NOVUS, Littleton, CO, USA). After antigen retrieval, sections were blocked with 1% bovine serum albumin (BSA) at RT for 1 h and then incubated with the primary antibody at 4 °C overnight, followed by specific fluorescent secondary antibody at RT for 1 h. All the sections were measured using a laser scanning confocal microscope (ZeissLSM510 Laser Module, Jena, Germany).

### 4.5. Cell Count

Sections stained with immunofluorescence were used for cell count. F4/80 and CCR7 double positive cells were identified as M1; F4/80 and CD206 double positive cells were designed as M2. The cells were counted in 5 independent visual fields from each sample under a high power (×400) randomly, and an average number was taken for analysis by SPSS16.0.

### 4.6. Western Blotting

The expression of Col-I, fibronectin, CD206 and TGF-β was examined by Western blotting. Visceral peritoneum tissue was lysed in 200 μL cell lysis buffer containing protease inhibitors for 10 min on ice. Then, the lysates were centrifuged at 12,500 rpm at 4 °C for 10 min, and the concentrations of the tissue protein were examined by a Microplate spectrophotometer (Molecular Devices, Silicon Valley, CA, USA). Samples with equal concentrations of tissue protein (20μg) were mixed with 2× sample buffer and heated at 95 °C for 10 min, then separated on 10% SDS-polyacrylamide gels. After electrophoresis for 2 h, the proteins were transferred onto a polyvinylidene fluoride (PVDF) membrane. The membranes were blocked with 5% non-fat milk at RT for 1 h and then incubated with the primary antibodies at 4 °C overnight, followed by specific peroxidase-conjugated secondary antibodies for 1 h at RT. After washing, the membranes were incubated with an enhanced chemiluminescence system (ECL) detection kit. Positive immunoreactive bands were normalized by glyceraldehyde phosphate dehydrogenase (GAPDH, CST, Boston, MA, USA).

### 4.7. Quantitative Real-Time PCR

Total RNA from peritonea was extracted using TRIzol reagent (Invitrogen, Carlsbad, CA, USA) and converted into cDNA using the Transcriptor First Strand cDNA Synthesis Kit (Roche, Basel, Switzerland), according to the manufacturer’s instructions. Amplification of specific PCR products was detected using the FastStart Universal SYBR Green Master (ROX) (Roche, Basel, Switzerland). Subsequently, the polymerase reactions were performed using the ABI Prism 7900HT (ABI, New York, NY, USA). Fold differences in gene expression were calculated as 2^−ΔΔCt^ using β-actin as the housekeeping gene [34]. Primer sequences used in this study are shown in Table 2.

### 4.8. Statistical Analysis

All numeric data were shown as the mean ± SD. Statistical analyses were performed by one-way ANOVA, with a LSD multiple comparison test using SPSS16.0 software (SPSS Inc., Chicago, IL, USA). *p <* 0.05 was considered statistically significant.

## 5. Conclusions

In conclusion, our study indicated that peritoneal M2 played an important role in the process of peritoneal fibrosis related to PD and might be a potential target for the intervention therapy of peritoneal fibrosis.

## Figures and Tables

**Table 1:** The thickness of peritoneum in four groups.

Group	Max (μm)	Min (μm)	Mean ± SD
NC	4.90	4.22	4.6 ± 0.34
PF	28.66	23.48	26.2 ± 2.03 #
LP	33.73	20.96	27.7 ± 4.38 #
LC	13.99	7.51	10.0 ± 2.36

# *p* < 0.05, *vs.* NC and LC group.

**Table 2:** Primer sequences for quantitative real-time PCR.

Gene	Primer sequence
β-actin	Forward: 5′-AGCCATGTACGTAGCCATCC-3′ Reverse: 5′-CTCTCAGCTGTGGTGGTG-3′
Col-I	Forward: 5′-TTTGGAGAGAGCATGACCGA-3′ Reverse: 5′-TGCTGTAGGTGAAGCGACTGTT-3′
Fibronectin	Forward: 5′-AATCACAGTAGTTGCGGCAGGAGA-3′ Reverse: 5′-TGTCATAGTCAATGCCAGGCTCCA-3′
Arg-1	Forward: 5′-AGAGCTGACAGCAACCCTGT-3′ Reverse: 5′-GGATCCAGAAGGTGATGGAA-3′
Ym-1	Forward: 5′-AATGATTCCTGCTCCTGT-3′ Reverse: 5′-ACTTTGATGGCCTCAACC-3′
